# Venous thromboembolism and radiation therapy: The final radiation‐induced thrombosis study analysis

**DOI:** 10.1002/cam4.4559

**Published:** 2022-02-24

**Authors:** Elisabeth Daguenet, Mathilde Maison, Fabien Tinquaut, Eric‐Alban Giroux, Laurent Bertoletti, Jean‐Philippe Suchaud, Chloé Rancoule, Jean‐Baptiste Guy, Nicolas Magné

**Affiliations:** ^1^ Département Universitaire de la Recherche et de l'Enseignement Institut de Cancérologie Lucien Neuwirth St‐Priest‐en‐Jarez France; ^2^ Département de Radiothérapie Institut de Cancérologie Lucien Neuwirth St‐Priest‐en‐Jarez France; ^3^ Service de Médecine Vasculaire et Thérapeutique CHU de St‐Etienne Saint‐Etienne France; ^4^ INSERM, UMR1059, Equipe Dysfonction Vasculaire et Hémostase Université Jean‐Monnet Saint‐Etienne France; ^5^ INSERM Saint‐Etienne France; ^6^ Département de Radiothérapie Centre Hospitalier de Roanne Roanne France

**Keywords:** ionizing radiation, *pan*‐cancer, prophylaxis, radiotherapy, venous thromboembolism

## Abstract

**Background:**

Thromboembolic events frequently complicate the course of malignancy and represent a major cause of morbidity and mortality in cancer patients. In contrast to chemotherapy and other systemic therapies, little is known about the impact of ionizing radiations on the incidence of venous thromboembolism (VTE) in cancer patients.

**Methods:**

In the present prospective study, we aimed to investigate the incidence, management, and outcome of VTE in newly diagnosed cancer patients who received curative radiotherapy.

**Results:**

VTE was found in 8 patients, out of 401 patients at a median time of 80 days after radiotherapy initiation. The incidence rate of VTE at 6 months post‐treatment was 2% (95% CI, 0.9–3.7), with 50% of cases occurring during the radiotherapy course and 50% of cases in patients who received or were receiving chemotherapy. As none of the patients harbored a personal history of VTE, no prophylactic measure was initiated during cancer therapy. Most patients received monotherapy with low‐molecular‐weight heparin and were still on surveillance at the end of the study. No specific clinical risk factor was identified that might systematically indicate the need of thromboprophylaxis in the context of curative radiotherapy.

**Conclusions:**

Although this *pan*‐cancer descriptive study did not relate an increased risk of short‐term thrombosis following ionizing radiation, it provides important insight as a basis for future studies with subcategories of cancer, in order to *in fine* guide further recommendations in frail patients.

**Clinical trial registration number:**

NCT02696447.

## Graphical abstract

This study investigates whether ionizing radiations may unfavorably influence treatment outcomes in a pan‐cancer population, by increasing the risk of venous thromboembolism in malignant patients. The present study shows that in pooled cancer patient populations, curative RT was not associated with an increased risk of VTE, when compared to other therapeutic strategies such as immunosuppressive or cytotoxic chemotherapy. Yet, the observations of the RIT study should be balanced to estimated annual incidences in which 0.5% of cancer patients will experience thrombosis compared with a 0.1% incidence rate in the general population. Overall, this study demonstrates that a careful assessment has to be done before RT to determine whether prophylactic measures are needed.

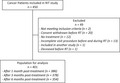

## 1 INTRODUCTION

Thrombosis in cancer is an important cause of morbidity and mortality, whose impact is significant on treatment, prognosis, and quality of life of cancer patients.^1^, ^2^ Thrombotic events can manifest from arterial or venous thromboembolism (VTE) to disseminated intravascular coagulation. Of note, the onset rate of VTE, which includes pulmonary thromboembolism and deep vein thrombosis, is four to seven times higher in cancer patients as compared to their noncancer counterparts.^3^, ^4^, ^5^ Epidemiologic studies have shown that 20%–30% of all first VTE events are cancer‐associated and that the cumulative incidence of VTE in cancer patients may reach to 8%.^2^, ^6^, ^7^ The pathobiology of cancer‐associated VTE is multifactorial and mainly implies the activation of coagulation and inflammatory pathways.^8^ Various risk factors have been described as contributing to VTE. Indeed, VTE incidence varies due to patient‐related factors (e.g. age, sex, comorbidities, and prior history of venous disease), tumor‐related factors (e.g. cancer localization, histology, stage), and treatment‐related factors (e.g. surgery, systemic chemotherapy, anti‐angiogenic treatment, hormonal and supportive therapies).^9^ Discerning factors associated with increased thrombosis in cancer is crucial in order to adequately identify patients who might benefit from thromboprophylaxis.

If anti‐neoplastic therapies or supportive care treatments have been shown to be associated with an increased risk of VTE, the impact of ionizing radiations per se on VTE has been less documented. Indeed, radiotherapy (RT) is a core modality for effective cancer treatment and control, either alone or in combination with systemic therapy, as it is estimated that around half of cancer patients would benefit from curative or palliative‐intent RT during their clinical course.^10^ To date, numerous studies have highlighted the pathogenic influence of ionizing radiations on endothelium activation and dysfunction, thus triggering long‐term risks of cardiovascular diseases, especially in patients receiving mediastinal RT.^11^, ^12^, ^13^, ^14^ Similarly, patients with head and neck malignancies receiving RT have a higher incidence of arterial stenosis through the formation of atherosclerotic plaques.^15^, ^16^ By contrast, RT is not considered as a classical VTE risk factor in cancer because its association with thromboembolic events has been barely evaluated in *pan*‐cancer cohorts. In a retrospective sub‐analysis of the RIETE registry, including 9284 patients with active cancer and VTE, there was a two‐fold higher risk for cerebral bleeding in patients treated with RT and concomitant anticoagulation therapy.^17^ Despite some evidences referring to the thrombogenic potential of RT^18^, ^19^, ^20^, no original study has thus far investigated the magnitude of increased risk of VTE during and following ionizing radiations. Therefore, the objectives of the herein study were to evaluate the incidence of VTE among cancer patients treated by ionizing radiations and to identify demographic‐ and disease‐related factors associated with VTE.

## 2 METHODS

### 2.1 Study design

**Figure 1:**
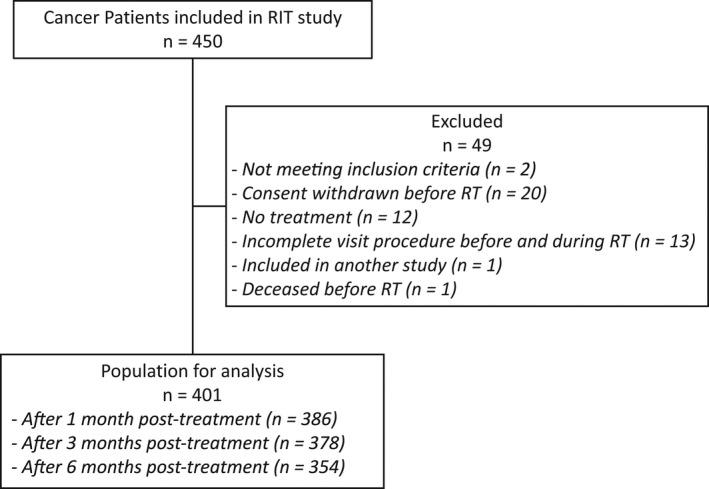
Flow diagram. RT, radiotherapy treatment

The RIT (for radiation‐induced thrombosis) study was an investigator‐initiated multicenter prospective trial, which started in June 2016 at the Institut de Cancérologie Lucien Neuwirth and at the Centre Hospitalier de Roanne (France). The study was approved by the institutional review board and was conducted in compliance with the international standards, including the International Conference on Harmonization (ICH) and the principles of the Declaration of Helsinki (NCT02696447). Adult patients (≥18 years) with a newly diagnosed malignancy or progressive disease requiring a treatment by RT or brachytherapy with curative intent were eligible for inclusion. Patients were not included if they presented a metastatic disease or if their follow‐up was not possible within the 6 month post‐inclusion. Furthermore, patients with an indication for long‐term therapeutic anticoagulation were excluded, but temporary treatment with low‐molecular‐weight heparin (LMWH) was allowed. In addition, patients on acetylsalicylic acid or other platelet inhibitors were not excluded. All patients gave their written consent and were prospectively followed for a maximum of 6 months, until loss of follow‐up, withdrawal of consent, or death. Until December 2019, 450 patients were included in this study. After re‐evaluation of the inclusion and exclusion criteria as well as the completion of at least one day of RT, 49 patients had to be excluded, because: (1) they did not fulfill inclusion or exclusion criteria (*n* = 2); (2) patients withdrew consent (*n* = 20); (3) no RT treatment was administered (*n* = 12); (4) no complete follow‐up during RT treatment was available (*n* = 13); (5) other reasons (*n* = 2). Thus, overall 401 patients were included in the analysis (Figure 1).

### 2.2 Outcome measurement

Venous thromboembolism is the primary outcome of RIT study during the observation time, defined as the occurrence of a thromboembolic event after at least one day of initiation of RT up to 6 months of the completion of RT course. This included any venous thrombosis and/or pulmonary embolism that were identified by clinical signs and diagnosis was confirmed by radiological imaging based on institutional guidelines (i.e., echo‐doppler, computerized tomography [CT], angiography or scintigraphy). An abjudication committee reviewed all events based on objective evidence. Asymptomatic event (e.g., incidentally detected event on CT scan) was considered an event if it was classified as clinically significant by members of the abjudication committee. Secondary outcomes included delay in the occurrence of VTE and association of thromboembolism with clinical variables.

### 2.3 Statistical analysis

With a confidence level of 95%, power of 90% and considering the prevalence of VTE at 3 months in cancer patients receiving chemotherapy to be around 3.4% ^21^ while the anticipated risk of developing VTE after ionizing radiations was 2‐fold increased, the minimum sample size to estimate a 3‐months VTE cumulative incidence of 6.0% was 450. Continuous variables were summarized as medians with interquartile range (IQR) and with ranges (minimum value – maximum value), and count data as absolute frequencies (%). The cumulative incidence of VTE was calculated using a competing risk estimator with 95% confidence intervals (95% CI). Univariate logistic regression analysis was performed to create odds ratios (ORs) and 95% CIs for classical VTE risk factors. Results with *p* values <0.05 were considered significant. All analyses were conducted using R statistical software, version 3.1.1 (R Foundation for Statistical Computing, Vienna, Austria).

## 3 RESULTS

### 3.1 Patients' demographics and clinical characteristics

The study cohort included 450 patients with a wide range of different cancer types. We analyzed data from 401 subjects who received at least one fraction of ionizing radiations; 66% (*n* = 265) were women, and 34% (*n* = 134) were men, with a median age of 65 years (range 33–88) and a median BMI of 25.4 kg/m^2^ (range 15.6–42.8). Table 1 shows the patients' characteristics of the RIT cohort as well as those of the evaluable patients at 1 month, 3 months and 6 months post‐treatment. The most common primary tumor site was breast (59.9%), followed by prostate (19.2%), and head and neck (10.2%) cancer (Table 2). Most participants (70.1%) had early disease and 83% had at least one comorbidity. Of note, less than 15% of patients had chronic respiratory disease or cardiac issues (*n* = 49 [12.2%] and *n* = 56 [14%], respectively); around 5% of patients had renal impairment or were diabetic (*n* = 18 [4.5%] and *n* = 26 [6.5%], respectively). While half of the population (*n* = 203) was active or ex‐smoker, a quarter of participants (*n* = 105) presented varicose veins and only 7.2% of patients (*n* = 29) had a personal history of VTE. At baseline, 30 patients (7.5%) were receiving thromboprophylaxis, with 28 patients treated with prophylactic‐intensity anticoagulation and 2 patients with LMWH (Table 1). Most patients (78.8%) underwent cancer surgery before radiation and 40% of patients also benefited from adjuvant or concomitant chemotherapy (Table 2).

**Table 1:** Characteristics of non‐VTE and VTE patients from the RIT cohort, at different time‐points following ionizing radiations

Timing (N. of patients)	RIT cohort (401)	1 Mo (382)	1 Mo + VTE (4)	3 Mo (370)	3 Mo + VTE (8)	6 Mo (346)	6 Mo + VTE (8)	*p‐value*
Clinical characteristics,								
Female gender	265 (66.1%)	256 (67.0%)	3 (75.0%)	248 (67.0%)	5 (62.5%)	234 (67.6%)	5 (62.5%)	0.72^¤^
Age, median (IQR), y	65 (56–70)	65 (56–70)	68 (65–70)	65 (56–70)	68 (65–77)	65 (56–70)	68 (65–77)	0.11^¤ ¤^
BMI, median (IQR), kg/m^2^	25.4 (22.2–29)	25.3 (22.1–28.9)	30.5 (29.2–30.8)	25.4 (22.1–28.9)	24.8 (23.7–30.5)	25.4 (22.2–28.8)	24.8 (23.7–30.5)	0.06^¤ ¤^
Underlying conditions,								
Chronic respiratory disease	49 (12.2%)	46 (12.0%)	1 (25.0%)	45 (8.2%)	1 (12.5%)	41 (11.8%)	1 (12.5%)	0.58^¤^
Moderate heart disease	56 (14.0%)	47 (12.3%)	‐	46 (12.4%)	‐	44 (12.7%)	‐	1^¤^
Renal disease	18 (4.5%)	17 (4.5%)	‐	17 (4.6%)	‐	16 (4.6%)	‐	1^¤^
Diabetes mellitus	26 (6.5%)	21 (5.5%)	‐	20 (5.4%)	‐	19 (5.5%)	‐	1^¤^
Prior history of cancer	53 (13.2%)	48 (12.6%)	1 (25.0%)	48 (13.0%)	1 (12.5%)	45 (13.0%)	1 (12.5%)	0.99^¤^
Risk factors for VTE,								
Recent surgery	55 (13.7%)	50 (13.1%)	‐	47 (12.7%)	‐	42 (12.1%)	‐	1^¤^
Immobility ≥4 days	14 (3.5%)	12 (3.1%)	‐	11 (3.0%)	‐	11 (3.2%)	‐	1^¤^
Recent travel ≥6 hours	3 (0.7%)	3 (0.8%)	‐	3 (0.8%)	‐	3 (0.8%)	‐	1^¤^
Prior VTE	29 (7.2%)	28 (7.3%)	‐	28 (7.6%)	‐	27 (7.8%)	‐	1^¤^
Varicose veins	105 (26.2%)	101 (26.4%)	2 (50.0%)	98 (26.5%)	5 (62.5%)	95 (27.5%)	5 (62.5%)	0.04^¤^
Obesity	62 (15.5%)	58 (15.2%)	3 (75.0%)	57 (15.4%)	3 (37.5%)	52 (15.0%)	3 (37.5%)	0.11^¤^
Smoking history	203 (50.6%)	189 (49.5%)	1 (25.0%)	184 (49.7%)	3 (37.5%)	171 (49.4%)	3 (37.5%)	0.72^¤^
Chronic ethylism	39 (9.7%)	33 (11.5%)	‐	27 (7.3%)	‐	27 (7.8%)	‐	1^¤^
Concomitant drugs,								
TE prophylaxis	30 (7.5%)	27 (7.1%)	‐	23 (6.2%)	‐	24 (6.9%)	‐	1^¤^
NSAIDs	36 (9.0%)	32 (8.4%)	‐	29 (7.8%)	2 (25.0%)	26 (7.5%)	2 (25.0%)	0.13^¤^

Abbreviations: IQR, interquartile; Mo, months post‐treatment; N., number; NSAIDs, non‐steroid anti‐inflammatory drugs; TE, thromboembolism; VTE, venous thromboembolism.^¤^ Fisher‐exact test; ^¤¤^ Wilcoxon test.

**Table 2:** Cancer characteristics of non‐VTE and VTE patients from the RIT cohort, at different time‐points following ionizing radiations

Timing (N. of patients)	RIT cohort (401)	1 Mo (382)	1 Mo + VTE (4)	3 Mo (370)	3 Mo + VTE (8)	6 Mo (346)	6 Mo + VTE (8)	*p‐value*
Site of cancer,								
Breast	240 (59.9%)	233 (61.0%)	2 (50.0%)	229 (61.9%)	2 (25.0%)	219 (63.3%)	2 (25.0%)	0.05^¤^
Prostate	77 (19.2%)	77 (20.2%)	‐	74 (20.0%)	2 (25.0%)	72 (20.8%)	2 (25.0%)	0.67^¤^
Head & Neck	41 (10.2%)	35 (9.2%)	1 (25.0%)	34 (9.2%)	1 (12.5%)	29 (8.4%)	1 (12.5%)	0.51^¤^
Cervix	11 (2.7%)	11 (2.9%)	‐	10 (2.7%)	1 (12.5%)	9 (2.6%)	1 (12.5%)	0.13^¤^
Gastrointestinal	15 (3.7%)	13 (3.4%)	‐	11 (3.0%)	1 (12.5%)	5 (1.4%)	1 (12.5%)	0.21^¤^
Lung	13 (3.2%)	10 (2.6%)	‐	9 (2.4%)	‐	9 (2.6%)	‐	1^¤^
Central nervous system	2 (0.5%)	2 (0.5%)	‐	2 (0.5%)	‐	2 (0.6%)	‐	1^¤^
Bladder	1 (0.2%)	‐	1 (25.0%)	‐	1 (12.5%)	‐	1 (12.5%)	0.02^¤^
Other	1 (0.2%)	1 (0.3%)	‐	1 (0.3%)	‐	1 (0.3%)	‐	1^¤^
Stage,								
Early	281 (70.1%)	272 (71.2%)	3 (75.0%)	263 (71.1%)	6 (75.0%)	247 (71.4%)	6 (75.0%)	0.99^¤^
Locally advanced	120 (29.9%)	110 (28.8%)	1 (25.0%)	107 (28.9%)	2 (25.0%)	99 (28.6%)	2 (25.0%)	0.99^¤^
Treatment for cancer,								
Surgery	316 (78.8%)	302 (79.1%)	3 (75.0%)	297 (80.3%)	3 (37.5%)	282 (81.5%)	3 (37.5%)	0.008^¤^
Chemotherapy	162 (40.4%)	149 (39.0%)	3 (75.0%)	143 (38.6%)	5 (62.5%)	131 (37.9%)	5 (62.5%)	0.27^¤^
Targeted therapy	39 (9.7%)	37 (9.7%)	‐	36 (9.7%)	‐	35 (10.1%)	‐	1^¤^
Hormonotherapy	221 (55.1%)	217 (56.8%)	2 (50.0%)	211 (57.0%)	3 (37.5%)	200 (57.8%)	3 (37.5%)	0.29^¤^
Radiotherapy,								
Median duration of treatment, days (IQR)	45 (43–49)	45 (43.2–49)	45 (42.8–47)	45 (44–49)	46.5 (43–51.8)	45 (44–49)	46.5 (43–51.8)	0.51^¤¤^
Median dose, Gy (IQR)	66 (60–66)	66 (60–66)	64.5 (63–66)	66 (60–66)	64.5 (60–66)	66 (60–66)	64.5 (60–69)	0.82^¤¤^
Median number of fractions (IQR)	33 (28–33)	33 (28–33)	31.5 (28.8–33.5)	33 (28–33)	31.5 (29.5–36)	33 (28.2–33)	31.5 (29.5–36)	0.77^¤¤^
Median CTV, cm^3^	430 (220–848)	429 (217–849)	736 (586–1084)	426 (213–812)	662 (438–834)	429 (211–812)	662 (438–834)	0.14^¤¤^
Median PTV, cm^3^	737 (415–1189)	737 (415–1184)	1308 (946–1662)	725 (400–1157)	1116 (430–1588)	720 (394–1154)	1116 (430–1588)	0.37^¤¤^

Abbreviations: CTV, clinical target volume; Gy, gray; IQR, interquartile range; Mo, months post‐treatment; N., number; PTV, planning target volume; VTE, venous thromboembolism.^¤^ Fisher‐exact test; ^¤¤^ Wilcoxon test.

### 3.2 Risk of VTE in patients with cancer after radiotherapy

During the follow‐up time, eight VTE events were observed in eight patients. Therefore, the 6‐month cumulative incidence rate of VTE was about 2% (95% CI, 0.9–3.7). VTEs included 7 (87.5%) deep vein thromboses (DVTs), with 3 (43%) in the lower limb and 4 (57%) in the upper limb, as well as 1 (12.5%) pulmonary embolism with DVT. VTE patients' characteristics are described in Table 3. The median time to VTE diagnosis after RT treatment was 80 days, ranging from 5 to 129 days. Noticeably, 4 events occurred during the time‐course of RT while others arose more than 100 days after treatment initiation. Relevant symptoms at VTE diagnosis included limb edema, and pain as well as elevated D‐dimers levels. Clinical and tumor characteristics were compared between patients with or without VTE in Tables 1 and 2 at 6 months post‐treatment. Varicose veins and cancer treatment with surgery were more frequent in patients with VTE compared with patients without VTE. These differences were statistically significant. Distribution of other characteristics was similar between the two groups. Interestingly, none of the VTE patients had a personal history of thrombotic events but some of them presented cardiovascular disease risk factors (4/8 patients) (e.g., hypertension, obesity) and varicose veins (4/8 patients) (Table 3). Of the 4 patients who received chemotherapy, 3 patients developed VTE during the radiotherapy course and 2 patients followed a concomitant chemo‐radiotherapy scheme (Table 3, Pt 2 & 6). The vast majority of the patients (n = 7, 87.5%) were treated with LMWH and one patient received a pentasaccharide factor Xa inhibitor. At 6 months post‐treatment, patients were still under surveillance and were pursuing their treatment. In order to identify clinical risk factors that might indicate the need of thromboprophylaxis, a univariate analysis was carried out and was not contributive due to the low effective sample size of the VTE group (data not shown). Therefore, no significant association between curative radiotherapy and the risk of VTE was found.

**Table 3:** Demographic and clinical characteristics and findings of VTE patients

Characteristics	Pt 1	Pt 2	Pt 3	Pt 4	Pt 5	Pt 6	Pt 7	Pt 8
Demographic characteristics,								
Age, y	79	67	80	67	76	56	58	67
Sex	Male	Male	Male	Female	Female	Female	Female	Female
Cancer site	Prostate	H&N	Prostate	Breast	Breast	Bladder	Cervix	Gastro‐intestinal
Medical history	Hypertension, rheumatoid arthritis	‐	Von Willebrand disease	Hypercholes‐ terolemia, chronic venous insufficiency	Hypertension	Hypothyroidism	‐	Hypertension
VTE risk factors	Varicose veins, hormonotherapy	Smoking history, chemotherapy	Varicose veins, smoking history	Varicose veins, hormonotherapy, obesity	Varicose veins, chemotherapy, obesity	Chemotherapy, obesity	Varicose veins, active smoker, chemotherapy	Chemotherapy
VTE characteristics,								
Days from RT to thrombotic event	115	32	129	45	5	43	117	126
VTE findings	PE with proximal DVT	Upper extremity DVT	Left lower extremity DVT, then bilateral DVT	Lower extremity SVT	Bilateral lower DVT	Upper extremity DVT	Upper extremity DVT	Upper extremity DVT
Symptoms at VTE onset	Elevated D‐dimers	‐	Edema	Pain, red cord in the subcutaneous tissue, elevated D‐dimers	Edema, elevated D‐dimers, dyspnea, pain	Edema	Edema, pain	Pain, inflammatory syndrome
VTE treatment	LMWH	LMWH	LMWH	Pentasaccharide	LMWH	LMWH	LMWH	LMWH

Abbreviations: DVT, deep vein thrombosis; H&N, head and neck; LMWH, low‐molecular weight heparin; PE, pulmonary embolism; Pt, patient; RT, radiotherapy; SVT, superficial vein thrombosis; VTE, venous thromboembolism; y, years.

## 4 DISCUSSION

The present study shows that in pooled cancer patient populations, curative RT was not associated with an increased risk of VTE, when compared to other therapeutic strategies such as immunosuppressive or cytotoxic chemotherapy.^2^, ^22^ During a follow‐up period of up to 6 months, 2.0% of patients developed VTE, essentially with a DVT presentation. These patients were not considered as high‐risk individuals, given that no thromboprophylaxis was initiated despite relevant medical history and/or the presence of VTE risk factors. Yet, the incidence in this study is much higher in comparison to the general population for which the estimated annual incidence of VTE was 184.0 per 100,000 subjects.^23^ Importantly, it is estimated that approximately 4%–20% of cancer patients will experience VTE at some stage during disease course. The observations of the RIT study should be balanced to estimated annual incidences in which 0.5% of cancer patients will experience thrombosis compared with a 0.1% incidence rate in the general population.^24^ One might also consider that the long‐term incidence could be underestimated given that around 8% of patients were lost‐to‐follow up at 6 months post‐treatment. Although this study did not show an increased risk of VTE during therapy course, it still demonstrates that a careful assessment has to be done before RT to determine whether prophylactic measures are needed.

Diagnosis and management of thrombotic events in cancer patients remains a major challenge for health care providers. In this regard, coagulation abnormalities may interrupt the treatment course and may expose to serious bleeding complications or VTE recurrence, thus contributing to a poor prognosis and a high disease‐specific mortality in cancer patients. The impact of ionizing radiations on VTE is a matter of debate and whether RT treatment per se favors the onset of VTE is uncertain. Indeed, discrepancies in its participation were noted in the literature, thus mitigating the etiologic role of RT in VTE development. For instance, a direct relationship has been highlighted in lung adenocarcinoma in which patients who had received RT were at higher risk of VTE compared to patients without RT (HR 2.1, 95% CI 0.6–7.1).^25^ In a small series, Guy and collaborators reported a plausible relationship between brachytherapy and the occurrence of VTE in patients with gynecological cancers.^18^ Moreover, in a retrospective analysis that stratified patients in three groups (i.e., RT for brain tumors, RT for body tumors, chemotherapy‐treated brain and body tumors), external beam RT was identified as an independent risk factor for VTE development in outpatient setting, with a risk difference of 5% (*p 0.018*) in comparison to chemotherapy.^26^ Similarly, in a recent sub‐analysis of the COMPASS‐CAT study, a significant correlation between RT and VTE was described in patients with breast, lung, ovarian or colon cancer (HR 2.47, 95% CI 1.47–4.12, *p 0.011*).^27^ Yet, other studies did not find any specific association between RT and cancer‐associated thrombosis. For example, in a large cohort of patients with prostate cancer, no link was established between curative RT and an increased risk of thromboembolic disease.^28^ Despite the description of a short‐term risk of VTE in rectal cancer, the analysis of the impact of preoperative RT based on the Swedish registry showed that the absolute rate of difference of VTE attributed to RT was low (10 cases per 1000 patients per year).^20^ So far, these differences among studies might be explained by population selection, in terms of tumor location. In fact, some cancer types are more prone to thromboembolic complications as defined by the Khorana predictive model, in which lung, lymphoma, gynecological, bladder or testicular cancers are classified as “high risk” whereas stomach and pancreas are considered as “very high risk”.^29^ Therefore, VTE incidence rate and the relationship to treatments, especially RT, may vary according to malignancy sites. Actually, when comparing the number of VTE events that were reported, VTE incidences upon RT are quite similar between studies and the present cohort. In the retrospective analysis that assessed VTE incidence in RT‐treated patients versus chemotherapy‐treated patients, four cases were reported in the RT group for body tumors, out of 158 patients, thus suggesting a comparable VTE incidence to our study.^26^ By contrast, in the post‐hoc analysis of the COMPASS‐CAT trial, the authors reported a higher VTE incidence of about 9.1%, with 33 events in 336 evaluable patients, considering that all patients were treated by chemotherapy and that 25% of patients were metastatic.^27^, ^30^ In this context, it is therefore tempting to speculate that the inclusion of advanced stage of cancers may bias this incidence, given that the presence of distant metastases increases the risk of VTE. ^5^, ^31^ Moreover, it is likely that an increased rate of VTE may be attributable to some degree to differences in baseline characteristics as well as to the presence of classical VTE risk factors in the studied populations. Estimating the true rate of VTE may be thus challenging in *pan*‐cancer patients. Our findings, in light with previous reports, emphasized that it is necessary to perform additional studies focusing on cancer subtypes to quantify in a precise manner frequency, risk factors, and impact on mortality of VTE in patients with specific diseases.

The risk of VTE in patients with cancer varies during the course of the cancer disease. It is highest during the first 6 months after diagnosis of cancer and then declines.^32^ Different guidelines cover the identification of patients at risk of VTE using risk assessment models, and indicate for prophylactic strategies or treatment. As long as most of the VTE events occur in the outpatient setting, primary thromboprophylaxis in ambulatory patients, especially upon ionizing radiations, may be beneficial but not recommended for all cancer patients, due to the uncertain benefit–risk balance associated with the risk of major bleeding.^33^, ^34^, ^35^ Findings from the RIETE registry indicated that patients with active cancer and VTE had an over two‐fold higher risk of cerebral bleeding during the course of anticoagulant therapy while receiving RT.^17^ Our study failed to identify specific VTE risk factors that might indicate a daily practice for some tumor entities. In their analysis, Temraz and collaborators revealed that breast cancer was at higher risk of VTE, conversely to colorectal cancer.^27^ Again, these controversial results demonstrated that no definite conclusion could be drawn. This further suggests that a patient risk stratification approach must be systematically applied to every patient before RT initiation. The Khorana score is the most widely used predictive score, based on five variables (cancer type, prechemotherapy platelet count, prechemotherapy hemoglobin level or use of red cell growth factors, prechemotherapy leucocyte count, and body mass index).^29^ To date, a dozen of risk scores has been derived from the Khorana score or novel original scores have been developed integrating other variables such as genetic factors.^33^ So far, none of these scores had reliably discriminated between patients at high risk and those at low‐ or intermediate‐risk for VTE in a specific cancer type or had been prospectively validated, in particular of the context of RT.^36^ To do so, a fine‐tuning in prediction tools, by incorporating biological dosages or machine learning‐driven approaches, is expected to better improve appropriate and safe use of VTE prophylaxis in cancer patients upon ionizing radiations.

## CONFLICT OF INTEREST

The authors have no competing interests.

## AUTHOR CONTRIBUTIONS

ED, MM, EAG, LB, CR, JBG and NM designed the research; ED, MM, JPS, CR, JBG and NM performed the research; ED, MM, JPS, CR, JBG and NM contributed to data collection; ED, MM, FT and NM analyzed the data; ED wrote the manuscript; ED, MM, FT, EAG, LB, JPS, CR, JBG and NM contributed to writing– review and editing of the manuscript.

## ETHICS STATEMENT

Approval of this study was obtained from the Ethics Committee of the institute. Written informed consents were obtained from all patients.

## Data Availability

The data that support the findings of this study are available on request from the corresponding author. The data are not publicly available due to privacy or ethical restrictions.
